# Awareness and Periodontal Health Practices of Fixed Orthodontic Appliance Patients: A Questionnaire-Based Survey

**DOI:** 10.7759/cureus.60335

**Published:** 2024-05-15

**Authors:** Kavitha O Marusamy, Rima B Alsibaie, Njoud M Mostanteq, Lamia Alzahrani, Dania H Aljuhani, Renad Lashkar

**Affiliations:** 1 Orthodontics and Dentofacial Orthopedics, Ibn Sina National College for Medical Studies, Jeddah, SAU; 2 Dentistry, Ibn Sina National College for Medical Studies, Jeddah, SAU

**Keywords:** knowledge, attitude, awareness, periodontal health, orthodontics

## Abstract

Abstract

A well-aligned dentition is more conducive to periodontal health, which is necessary for successful orthodontic therapy. Maintaining good dental hygiene is crucial for effective treatment, and patient cooperation, education, motivation, and attitude are all important components. Orthodontists must routinely check in with their patients to see whether they are maintaining their oral hygiene and if they are using any additional assistance. Negligence on the part of the patient may be the cause of poor treatment outcomes. This study aims to show how patient education can affect treatment outcomes and the development of a functional, aesthetically pleasing, and healthy dentition.

Aim

This study aims to investigate awareness, attitude, and periodontal health knowledge among orthodontic patients.

Objectives

The study explores the level of awareness and knowledge regarding periodontal health among orthodontic patients, examining its correlation with factors such as age, attitude, and duration of orthodontic treatment. Additionally, it aims to gauge the extent of education received by orthodontic patients regarding proper oral hygiene practices and the potential repercussions of neglecting them.

Methods

A questionnaire-based study with a cross-sectional design was performed in Jeddah, Saudi Arabia. A total of 428 participants were randomly selected from several private orthodontic clinics and hospitals. The participants in the study were individuals currently undergoing treatment with fixed orthodontic appliances. Information was gathered using a self-administered questionnaire.

Results

The patients undergoing orthodontic treatment have a moderate understanding of periodontal disease about dental plaque. The level of awareness of periodontal health was 41%, and the level of knowledge about periodontal health was 51%. When it came to periodontal health, adult orthodontic patients had a positive attitude toward fixed orthodontic treatment (mean score = 0.75). Subjects' attitudes regarding fixed orthodontic treatment were significantly impacted by the patient's consistency with his/her dental checkups (p value = 0.02).

Conclusion

The patients’ periodontal health awareness was moderate, while their periodontal health knowledge was fair. Orthodontic patient's awareness levels, as well as knowledge levels, showed significant differences in age and duration but not sex. Results showed no significant difference among the attitude levels of orthodontic patients and age, sex, and duration.

## Introduction

Dental malocclusion with misalignment of teeth and periodontal disease show a notable correlation. Malocclusion fosters plaque accumulation, heightening the risk of periodontal disease. Treating malocclusion is vital for oral health maintenance. Moreover, it is undisputed that good periodontal health is integral to successful orthodontic therapy [1]. Although the positive effects of orthodontic treatment on periodontal health are supported by many studies, current evidence suggests that orthodontic therapy may still result in detrimental effects on periodontal health [2]. Adequate oral hygiene instructions should be conveyed to prevent dental complications including caries, gingivitis, and periodontitis during fixed orthodontic treatment. Such complications are caused by the fixed appliance's nature to reduce saliva fluidity, affecting saliva's lubrication and protective effect on teeth, which increases the incidence of caries. Additionally, fixed orthodontic appliances tend to have several plaque retentive areas, which makes it more difficult for patients to properly maintain their oral environment plaque-free [3]. Instructions for proper oral hygiene can be portrayed in different manners (i.e., educational videos, demonstrations on a mannequin, brochures, or even as simple as rationally talking to the patient).

Orthodontists must check with the patient routinely about their oral hygiene maintenance and ensure their use of additional oral hygiene aids that help keep the treatment plan on track. The consequences of failing to do so can only aid in the progress of disease, further tissue degeneration, and worsen periodontal complications such as gingivitis, periodontitis, gingival recession or hypertrophy, and alveolar bone loss that would perplex the results of the treatment [4]. Therefore, awareness and patient education are compulsory because in the end their motivation and consistency with their oral hygiene regimen are fundamental for successful orthodontic treatment. Inadequate maintenance could stem from either a deficiency in understanding or negligence on the part of the patients themselves [5]. Several studies have highlighted the subpar awareness among orthodontic patients regarding their gingival health, even after receiving comprehensive guidance from their orthodontic specialists. Consequently, a significant number of patients tend to overlook these instructions and underestimate their significance [6].

While some studies suggest that certain malocclusions, such as deep overbites or open bites, may contribute to gum recession or periodontal disease because of improper distribution of biting forces, other studies have not found a significant association between malocclusions and periodontal health problems [7,8]. Furthermore, it has been reported that orthodontic patients’ awareness of their periodontal health is poor and can be affected by multiple factors such as age, attitude, and treatment duration [9]. Hence, the primary objective of this study is to assess periodontal health awareness and practices within the cohort of fixed orthodontic patients, with a secondary aim to elucidate potential correlations between demographic factors such as age, gender, and educational qualification.

## Materials and methods

Ethical committee approval for this study was obtained from the Ibn Sina National College Institutional Research Review Board (ISNC-IRRB Approval no 005DB28012024). A random sampling technique was adopted in the present study. With an approximate population of 10,000 orthodontic patients in the Jeddah region, a confidence level of 95%, a margin of error of 5%, and a response distribution of 50%, the recommended sample size calculated is 370, but the data of 428 patients were collected. A structured questionnaire was distributed to qualified orthodontic patients enlisted from orthodontic clinics situated in private clinics and hospitals. Those currently undergoing orthodontic treatment at the clinics during the study period and consented to participate were incorporated into the study. Both verbal and written explanations about the study were provided to the patients. Additionally, all participants belonged to comparable economic and social backgrounds.

Approximately 428 orthodontics patients (274 males and 154 females) participated in this study. The patient’s age was divided into two groups (more than 18 years of age and less than 18 years of age). Educational level was recorded, which ranged from intermediate education to master's. Subjects were currently wearing fixed orthodontic appliances for an average duration of 12 months. The treatment duration ranged from less than 12 months to more than 18 months. Before commencing orthodontic treatment, all participants exhibited healthy periodontal conditions and were educated by their orthodontists about the importance of thorough dental hygiene following the appliance placement. Exclusions comprised individuals with cognitive impairments, chronic medical conditions, craniofacial abnormalities such as cleft lip and palate, and those diagnosed with aggressive periodontitis. Additionally, the study did not include patients receiving treatment from government health facilities.

The data collection instrument utilized in this study was a self-administered structured questionnaire, previously employed in a study conducted by Alhaija et al. [9]. Pilot data of 50 patients were collected and analyzed. The kappa statistic value was calculated to be 85%. Then the questionnaire was ready and distributed among sample participants. Participants completed the questionnaire while waiting in the orthodontic clinic's waiting areas.

The first part of nine dental health questions inquired about demographic details such as age, duration of fixed orthodontic treatment, and current oral health habits (including frequency and duration of toothbrushing, use of auxiliary aids, and regularity of dental visits). Six questions focused on the participants' awareness of periodontal health, with response options including "yes," "no," and "I don’t know." Additionally, questions related to periodontal knowledge covered topics such as dental plaque, its consequences, interpretation of bleeding gums, and methods to prevent gum disease. Responses were structured as multiple choice, with only one correct answer provided. Other inquiries assessed the patients' overall knowledge of oral and periodontal health and the sources of their information. Subjects' awareness of orthodontic treatment and its relationship with periodontal health was gauged, with scores assigned as zero for negative responses and one for positive ones. Based on their cumulative scores, participants were categorized into groups reflecting either a positive attitude (average score: 6-9) or a negative attitude (average score: 0-5) toward periodontal health and orthodontic treatment.

## Results

There were 428 patients who participated in the study. The majority 322 (75.2%) were aged more than 18 years, while 106 (24.8%) were less than 18 years old. Of the participants, 274 (64%) were male and 154 (36%) were female. In terms of education, the majority 250 (58.4%) had a bachelor's degree, 99 (23.1%) had high school education, 39 (9.1%) had a diploma, 22 (5.1%) had a postgraduate degree, and 18 (4.2%) had an intermediate level of education.

The results of the routine dental health questionnaire (Table 1) showed that the majority 323 (75.55%) were wearing fixed orthodontic appliances for less than 12 months, and 105 (25.5%) for more than 12 months. The majority 232 (54.2%) were brushing their teeth two to three times per day, 118 (27.6%) more than three times per day, and 78 (18.2%) once per day. While comparing the duration of brushing, the majority 248 (57.9%) brushed for less than three minutes, 140 (32.7%) from three to five min, and 40 (9.3%) brushed for more than five minutes. As for the type of toothbrush, 59.6% used a regular toothbrush, 40.4% used an orthodontic toothbrush, 40.4% brushed their teeth horizontally, and 39.7% brushed randomly. Approximately 83.9% of patients were using auxiliary aids, 16.1% were not, and most of them (49.5%) were using dental floss, 30.4% were using an interdental brush, and 20.1% were using toothpicks. Specifically, 279 (64.5%) of them visited the dentist regularly before having orthodontic treatment, and 149 (35.5%) did not. Simultaneously, 65.2% visited the dentist regularly after wearing the orthodontic appliance, a marginal increase.

**Table 1:** Routine dental health measures questionnaire The data have been represented as N, %.

Dental health questions	N	%
How long have you been wearing an orthodontic appliance		
Less than 18 months	323	75.5
More than 18 months	105	24.5
Frequency of toothbrushing (time/day)		
1-2	78	18.2
2-3	232	54.2
>3	118	27.6
Duration of toothbrushing in minutes		
<3	248	57.9
3-5	140	32.7
>5	40	9.3
Type of toothbrush		
Orthodontic toothbrush	173	40.4
Regular toothbrush	255	59.6
Method of brushing		
Horizontal	174	40.7
Random	170	39.7
Other	84	19.6
Using auxiliary aids		
No	69	16.1
Yes	359	83.9
Type of auxiliary aids		
Dental floss	212	49.5
Toothpick	86	20.1
Interdental brush	130	30.4
Before wearing a fixed appliance, did you visit the dentist regularly for checkups?		
No	152	35.5
Yes	276	64.5
After wearing a fixed appliance, did you visit the dentist regularly for checkups?		
No	149	34.8
Yes	279	65.2

The level of awareness of periodontal health (Table 2) was 41%, and the most common symptom among participants was stains on teeth surfaces and gingival bleeding, followed by dental calculus and plaque on teeth surfaces, and gingival pain and irritation.

**Table 2:** Patient's awareness about periodontal health The data have been represented as mean±SD.

No	Awareness of periodontal health questions	Mean	± SD
1	Do you have dental plaque on your teeth surfaces?	0.40	0.49
2	Do you have dental calculus on your teeth surfaces?	0.43	0.50
3	Do you have stains on your teeth?	0.48	0.50
4	Do you feel gingival irritation?	0.39	0.49
5	Do you feel gingival pain?	0.36	0.48
6	Do you have gingival bleeding?	0.41	0.49
	Total (41%)	0.41	0.36

The findings revealed a favorable disposition toward periodontal health, with a mean score of 0.75±0.23 (Table 3). Participants exhibited a positive inclination toward maintaining oral hygiene practices, particularly in terms of toothbrushing following the placement of orthodontic appliances, and demonstrated a strong belief in the importance of adhering to the advice and instructions provided regarding oral hygiene by the clinicians, with a mean score of 0.92.

**Table 3:** Attitude toward periodontal health The data have been represented as mean±SD.

No	Attitude toward periodontal health questions	Mean	± SD
1	Fixed orthodontic appliance initiate/cause periodontal problems	0.38	0.48
2	Fixed orthodontic appliance makes brushing more difficult	0.75	0.44
3	Fixed orthodontic appliance causes severe pain	0.56	0.50
4	Straight teeth are easier to clean	0.87	0.36
5	It is important to get advice and instructions in oral hygiene from clinicians	0.89	0.32
6	It is important to follow the advice and instructions in oral hygiene given by clinician	0.92	0.27
7	It is important to brush more after wearing a fixed orthodontic appliance	0.92	0.28
	Total	0.75	0.23

The results of factors affecting the awareness, knowledge, and attitude toward periodontal health (Table 4) showed that there was a significant difference in the awareness level according to age (t = 5.046, p value < 0.001), education (F = 3.294, p value = 0.011), frequency of toothbrushing (F = 3.661, p value = 0.027), duration of toothbrushing (F = 8.364, p value < 0.001), method of brushing (F = 3.518, p value = 0.031), visiting the dentist regularly for a checkup before wearing a fixed appliance (t = -3.604, p value < 0.001) and visiting the dentist regularly for a checkup after wearing fixed appliance (t = -3.859, p value < 0.001), but there was no significant difference according to sex, duration of wearing orthodontic appliances, type of toothbrush, using of auxiliary aids, and type of auxiliary aids.

**Table 4:** Variables affecting the awareness, knowledge, and attitude toward periodontal health The data have been represented as T-value and p value, with a p value < 0.001.

Variables	Categories	Awareness	Knowledge	Attitude
Age	Less than 18 years	0.58	0.46	0.77
	More than 18 years	0.36	0.53	0.75
	T	5.046	-2.314	0.920
	P value	<0.001	0.021	0.358
Sex	Male	0.4	0.51	0.75
	Female	0.43	0.5	0.76
	T	-0.911	0.448	-0.621
	P value	0.363	0.654	0.535
Education	Intermediate	0.3	0.43	0.75
	High school	0.51	0.47	0.74
	Diploma	0.42	0.49	0.76
	Bachelor	0.39	0.53	0.76
	Postgraduate	0.27	0.61	0.76
	F	3.294	2.118	0.167
	P value	0.011	0.078	0.955
How long have you been wearing an orthodontic appliance	Less than 18 months	0.29	0.52	0.7
	More than 18 months	0.43	0.54	0.77
	t	-0.500	-1.395	-0.954
	P value	0.617	0.165	0.358
Frequency of toothbrushing (time/day)	1 to 2	0.5	0.45	0.76
	2 to 3	0.37	0.55	0.76
	>3	0.43	0.48	0.73
	F	3.661	5.329	0.662
	P value	0.027	0.005	0.615
Duration of toothbrushing in minutes	<3	0.4	0.5	0.76
	3 to 5	0.38	0.55	0.74
	>5	0.63	0.43	0.75
	F	8.364	3.560	0.727
	P value	<0.001	0.029	0.484
Type of toothbrush	Orthodontic toothbrush	0.41	0.52	0.76
	Regular toothbrush	0.41	0.5	0.75
	T	-0.143	0.510	0.403
	P value	0.883	0.611	0.687
Method of brushing	Horizontal	0.39	0.51	0.73
	Random	0.47	0.51	0.77
	Other	0.35	0.51	0.76
	F	3.518	0.008	1.232
	P value	0.031	0.992	0.293
Using auxiliary aids	No	0.39	0.39	0.68
	Yes	0.42	0.53	0.77
	T	-0.507	-4.081	-2.940
	P value	0.612	<0.001	0.003
Type of auxiliary aids	Dental floss	0.43	0.53	0.76
	Toothpick	0.44	0.48	0.75
	Interdental brush	0.36	0.49	0.74
	F	1.977	1.516	0.198
	P value	0.140	0.221	0.821
Before wearing the fixed appliance, did you visit the dentist regularly for a checkup?	No	0.33	0.49	0.72
	Yes	0.45	0.52	0.78
	T	-3.604	-0.915	-2.587
	P value	<0.001	0.361	0.010
After wearing the fixed appliance, did you visit the dentist regularly for a checkup?	No	0.33	0.51	0.72
	Yes	0.46	0.51	0.77
	T	-3.859	-0.142	-2.350
	P value	<0.001	0.887	0.019

There was a significant difference in the knowledge level according to age (t = -2.314, p value = 0.021), frequency of toothbrushing (F = 5.329, p value = 0.005), duration of toothbrushing in minutes (F = 8.364, p value <0.001), using of auxiliary aids (t = -4.081, p value < 0.001), but there was no significant difference according to sex, education, duration of wearing orthodontic appliances, type of toothbrush, type of auxiliary aids, method of brushing, visiting the dentist regularly for checkup before wearing a fixed appliance, and visiting the dentist regularly for checkup after wearing a fixed appliance. There was a significant difference in the attitude level according to using auxiliary aids (t = -2.940, p value = 0.003), visiting the dentist regularly for checkups before wearing a fixed appliance (t = -2.587, p value = 0.010), and visiting the dentist regularly for a checkup before wearing a fixed appliance (t = -2.350, p value = 0.019), but there was no significant difference according to age, sex, education, duration of wearing orthodontic appliances, frequency of toothbrushing, duration of toothbrushing, type of toothbrush, method of brushing, and type of auxiliary aids.

## Discussion

The study offered a thorough examination of the understanding, attitude, and awareness of orthodontic patients concerning their periodontal health. For the sample of orthodontist patients, there was a 2:1 male-to-female ratio. According to the statistical analysis of our study's respondents, there was no significance about sex (p value > 0.05). Other authors found a 1:3 male-to-female ratio [9]. The oral hygiene habits of patients in this study were good. 54.2% brushed their teeth two to three times per day, 57.9% brushed for less than three minutes and almost 60% brushed with a normal toothbrush. This was expected since around 65% of patients were regularly visiting their orthodontist before and after wearing fixed orthodontic appliances. Alhaija et al. [9]. showed that the oral hygiene behaviors of participants in their study were good. Other authors suggested that routine dental visits may have a positive effect on laypersons' knowledge about periodontal health [10]. However, Atassi et al.'s [11] study results showed that 40% of the patients had fair oral hygiene, whereas the majority of them had poor oral hygiene 60%. Only 32% of the participants reported visiting the dental hygienist during their orthodontic treatment, while the remaining 68% did not.

The level of awareness of periodontal health among patients was 41%, and the most common symptom among participants was gingival pain. Other complaints include gingival irritation, followed by the presence of dental plaque on teeth surfaces, gingival bleeding, the presence of dental calculus on teeth surfaces, and the presence of stains on teeth. Lie et al. [12] found in their study that 68% of the patients were not aware of the existing periodontal disease, and 74% had never received any information about the treatment possibilities. Ninety percent claimed that they had never received any periodontal treatment at all. The level of knowledge about periodontal health was 51%, the most source of information about oral and periodontal health was from a physician, and a positive attitude toward periodontal health with a mean score of 0.75. Baheti et al. [13] showed that nearly half of their study patients were unaware of periodontal health.

Elbelkemy et al. [14] conducted a hospital-based cross-sectional study to assess periodontal health knowledge among patients with fixed orthodontic appliances. The findings underscored varying levels of awareness among participants, highlighting the need for targeted educational interventions within orthodontic settings to enhance periodontal health literacy and promote better oral hygiene practices [14]. Another study about oral hygiene awareness and practices among orthodontic patients in Makkah City revealed discrepancies in oral hygiene behaviors. Despite the high prevalence of orthodontic treatment, there were notable gaps in oral hygiene knowledge and adherence among participants. This emphasizes the importance of comprehensive patient education initiatives to address these gaps and improve oral health outcomes in orthodontic patients [15]. Priyadarsi et al.'s study focused on assessing awareness about periodontal health among patients with fixed orthodontic appliances. The results indicated a lack of comprehensive understanding of periodontal health concepts among the study population [16]. This underscores the significance of integrating periodontal health education into orthodontic treatment protocols to empower patients with the knowledge necessary for optimal oral health maintenance throughout their treatment journey.

Addressing knowledge gaps and improving oral hygiene practices among orthodontic patients is crucial for enhancing periodontal health outcomes and overall oral health status within this population. The study revealed that orthodontic patients possess limited knowledge but moderate awareness of periodontal health, emphasizing the need for enhanced oral health education. With consistent efforts, patients can successfully complete their fixed appliance treatment while safeguarding their periodontal health. Orthodontists play a pivotal role in educating patients about periodontal health and promoting effective oral hygiene practices as part of long-term treatment protocols. Additionally, self-directed educational materials, such as pamphlets, YouTube, and social media offer practical and cost-effective means to encourage individuals to consider beneficial changes in their oral health habits. The study findings indicated significant differences in awareness levels based on factors such as age, education, frequency and duration of toothbrushing, brushing methods, and regular dental checkups before and after wearing a fixed appliance, while no significant differences were observed based on gender, duration of wearing orthodontic appliances, and types and use of toothbrushes and auxiliary aids.

Artificial intelligence (AI) chatbots can revolutionize periodontal health support for orthodontic patients. AI chatbots can provide patients with easily accessible information about periodontal health, including preventive measures, treatment options, and proper oral hygiene practices. They can answer frequently asked questions and address common concerns, empowering patients to take better care of their oral health. Chatbots can send automated reminders to patients for their upcoming dental appointments, reducing no-show rates and improving patient adherence to treatment plans. AI chatbots equipped with natural language processing (NLP) capabilities can engage in interactive conversations with patients. Patients can describe their oral health concerns to the chatbot, which can then offer guidance on whether immediate attention is needed or if the issue can be managed at home [17].

Study limitations

The limitations of this study encompass a relatively homogenous sample that may not accurately represent the entire population's characteristics or provide sufficient statistical power to detect significant effects. Further, varying female-to-male gender distribution within the study sample can introduce bias or confounding factors. This study also included subjects with diverse education levels. The differences in education levels among participants may influence their understanding of oral health concepts, compliance with treatment protocols, and overall outcomes. Study patients came from different socioeconomic backgrounds, and may have varying access to healthcare resources, oral hygiene practices, and nutritional habits, which can confound the study results and limit the generalizability of findings. Finally, the subjects were recruited strictly from private orthodontic practices and hospitals, which may introduce selection bias, as these patients may differ from those treated in public or community-based settings in terms of socioeconomic status, severity of dental conditions, and access to care. The duration and success of the interventions provided to the study participants were not measured. This includes any modifications of orthodontic treatment, periodontal care, and any other interventions aimed at improving oral health outcomes. 

Increasing the sampling relevance by collaborating with multiple orthodontic practices, diversifying recruitment to include public healthcare settings, extending study duration, stratifying samples based on demographics, and standardizing the recruitment criteria can minimize bias. Accounting for confounders with comprehensive data collection and statistical techniques will increase the reliability of this study.

## Conclusions

The present study highlights the need to improve awareness regarding periodontal health issues among patients with fixed orthodontic appliances. The knowledge and awareness of periodontal health practices were better among younger age group individuals. However, targeted educational efforts within orthodontic settings are crucial for enhancing awareness and promoting effective oral hygiene practices.

## Appendices

**Table 5:** Complete study questionnaire

1-Age	
2-Gender	Male/Female
3-Level of education	
4-How long have you been wearing orthodontic appliances? (months)	
5-Frequency of toothbrushing per day (times/day)	No brushing/<1/1-2/>3
5-Duration of toothbrushing in minutes	<3/3-5/>5
6-Type of toothbrush	Orthodontic toothbrush/Ordinary toothbrush
7-Method of brushing	Vertical/Horizontal/Haphazard/Other
8-Before wearing a fixed appliance, did you visit the dentist regularly for checkups?	Yes/No
9-After wearing a fixed appliance, did you visit the dentist regularly for checkups?	Yes/No
10-Do have dental plaque or biofilm on your teeth surfaces?	Yes/No/I don’t know
11-Do you have dental calculus on your teeth surfaces?	Yes/No/I don’t know
12-Do you have stains on your teeth?	Yes/No/I don’t know
13-Do you have gingival irritation?	Yes/No/I don’t know
14-Do you feel gingival pain?	Yes/No/I don’t know
15-Do you have gingival bleeding?	Yes/No/I don’t know
16-Have knowledge and information about oral and gum health ?	Yes/No
17-If yes, from where did you get your information	Dentist/School/Other
18-Do you know what dental plaque is?	Soft deposit on tooth/Hard deposit on tooth/Stains/I don’t know
19-Do you know what bleeding gums indicate?	Healthy gums/Inflamed gums/Gum recession/I don’t know
20-Do you know how to prevent gum disease?	Brushing/Diet control/Vitamin intake/I don’t know
21-Does fixed braces increase gingival inflammation?	Yes/No/I don’t know
22-Does fixed braces make brushing more difficult?	Yes/No
23-Does fixed braces cause severe pain?	Yes/No
24-Are straight teeth easier to clean?	Yes/No
25-Is it important to get advice and instruction in oral hygiene given by the clinician?	Yes/No
26-Is it important to brush more after wearing a fixed orthodontic appliance	Yes/No
27-Is it important to follow the advice and instructions in oral hygiene from the clinician?	Yes/No
